# A Formal and Quantifiable Log Analysis Framework for Test Driving of Autonomous Vehicles

**DOI:** 10.3390/s20051356

**Published:** 2020-03-02

**Authors:** Kyungbok Sung, Kyoung-Wook Min, Jeongdan Choi, Byung-Cheol Kim

**Affiliations:** 1 Autonomous Driving Intelligence Research Section, Artificial Intelligence Research Laboratory, Electronics and Telecommunications Research Institute, Daejeon 34129, Korea; kbsung@etri.re.kr (K.S.); kwmin92@etri.re.kr (K.-W.M.); jdchoi@etri.re.kr (J.C.); 2 School of Software Engineering, Joongbu University-Goyang, Goyang 10279, Korea

**Keywords:** autonomous vehicle testing, log analysis framework, failure detection, formal methods, metamorphic testing

## Abstract

We propose a log analysis framework for test driving of autonomous vehicles. The log of a vehicle is a fundamental source to detect and analyze events during driving. A set of dumped logs are, however, usually mixed and fragmented since they are generated concurrently by a number of modules such as sensors, actuators and programs. This makes it hard to analyze them to discover latent errors that could occur due to complex chain reactions among those modules. Our framework provides a logging architecture based on formal specifications, which hierarchically organizes them to find out a priori relationships between them. Then, algorithmic or implementation errors can be detected by examining a posteriori relationships. However, a test in a situation of certain parameters, so called an oracle test, does not necessarily trigger latent violations of the relationships. In our framework, this is remedied by adopting metamorphic testing to quantitatively verify the formal specification. As a working proof, we define three metamorphic relations critical for testing autonomous vehicles and verify them in a quantitative manner based on our logging system.

## 1. Introduction

The autonomous vehicle (AV) industry is growing fast thanks to the improvement of artificial intelligence (AI) technologies based on sensing devices [1,2,3]. By now, many AVs from tens of companies are being driven in real road environments for safety and performance tests [4,5,6]. Some of them have been driven tens of million of miles, and even billions in simulations. There are also statistical analyses on how many miles are needed to examine the safety level of AVs [7]. However, the approaches have mainly focused on the number of miles driven, not on how those miles are examined.

Extended miles need long, continuous, and/or repetitive engagement of human observers who get easily tired of such routine tasks. They would often lose concentration on critical details, which is the objective to be sought after, and cannot prevent a fatal accident even though they are in the driver’s seat [8]. Thus, inspectable automation tests are needed, and this requires logging to automatically collect all the necessary information to be examined.

How can we test for latent errors in the formidable heap of log data? The conundrum in examining intelligent machines basically comes from the property of its software. As the software engineering field had already elucidated its fundamental difficulty, it is an interpolation problem that one cannot conclude the interval is as good as the ends only from end-point tests. For instance, if a motor works properly at 100 revolutions per minute (RPM) and 10,000 RPM, then we can expect it will also work well from 100 to 10,000 RPM. A software program, however, does not allow this kind of interpolation in testing. Trivially, one ‘if’ statement could ruin the interpolation. Thus, we should test them not only at each gate condition but also in the parameter span as exhaustively as possible.

Since this needs infinite tests, theoretically, various logical approaches have been suggested [9,10] to find out the optimal set of tests of reasonable size. The formal method, for example, hierarchically decomposes the parameter space into subcategories of manageable size in a top-down manner (i.e., formal specification) and extract meaningful combinations of them for testing (i.e., formal verification). However, tests of these combinations with specific parameter values are variants of the oracle test, which is, in turn, a sophisticated version of end-point tests. We still do not know what will happen outside the designated values of parameters in the test case.

To alleviate the oracle test problem, a mathematical approach, so-called metamorphic testing (MT), has been suggested [11,12]. It converts metamorphic relations (MRs), mathematically definable relationships that separate sets of data into quantifiable data and arithmetically checks the values to examine whether there are violations of the relationships. Recent applications of MT to AV testing have shown promising results and have discovered unknown errors in the AV modules [13].

MRs that the state-of-the-art MT for AVs are based on are, however, defined case by case, which might look like another version of oracles. Our observation is that MRs for AVs can be extracted from logical relationships defined using formal specification. Then, if the logging architecture takes into account both formal specifications and corresponding metamorphic relations, it could fundamentally support inspectable automation testing. In addition, it should be able to be used interchangeably in both real and simulated vehicles to make simulation tests as valuable as real tests, which are more expensive, dangerous, and time-consuming.

**Figure 1:**
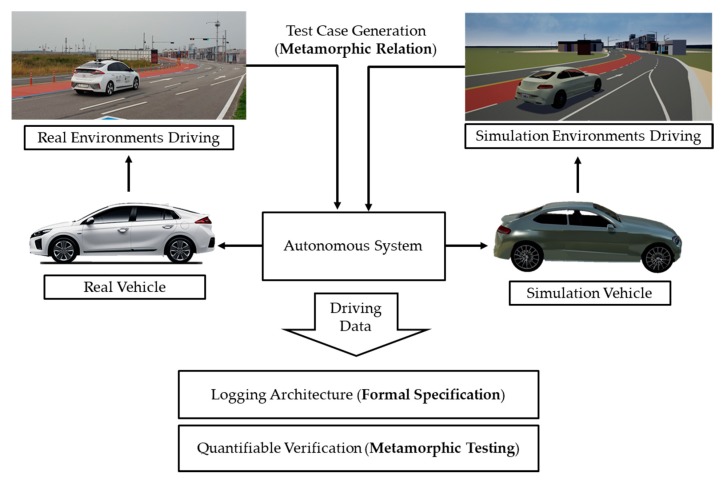
Overview of a formal and quantifiable log analysis framework.

Therefore, we propose a formal and quantifiable log analysis framework for test driving of AVs, incorporating complementary virtues of each methodology mentioned above. Figure 1 shows a log analysis framework. In the next subsections, we review the relevant topics in more detail.

### 1.1. Driving Data Recording

Efforts to record the state of vehicle driving have been made before. An event data recorder (EDR) is a data recording device that has been installed in vehicles since the late 1990s to identify the driving state and the cause of the accidents. Many institutions recommend data items that EDR should record. The National Highway Traffic Safety Administration (NHTSA) proposes to record the signals of longitudinal acceleration, lateral acceleration, yaw rate, engine RPM, brake, and turns. Although the items of the Society of Autonomous Engineers (SAE) are similar to the NHTSA’s, except the yaw rate, it also suggests to record seatbelt use.

In some areas, experiments on public roads for autonomous driving research are authorized. In addition, it is mandatory to install a data recorder that stores the information of the autonomous vehicle. In the case of California, it is also required to install a separate data recorder based on the California Code of Regulations [14]. In Korea, a data recorder is obligatory as a basic requirement to obtain a temporary license for autonomous driving on public roads [15]. The data recorder must save these items: the operation mode, brake and acceleration pedal use, steering wheel angle, and gear position. In addition, speed, acceleration, front and rear video footage, and indoor video footage must be stored too.

Recently, due to the development of deep learning technology, there has arisen an effort to record all the data of a vehicle. Datasets such as the KITTI dataset [16], Cityscape dataset [17], etc., include all sensor data and vehicle driving information and can be used for deep learning. These data sets include not only the location, speed, acceleration of the vehicle but also image information obtained from the camera, a Light Detection and Ranging (LiDAR) sensor, and GPS. This information is very detailed and includes not only the ego-vehicle but also the information of the environment surrounding the vehicle, which is very useful to analyze autonomous driving problems. However, these data are so large that they take up a lot of storage and are difficult to analyze. As a result, there are difficulties in testing various environments.

### 1.2. Formal Methods

Formal methods include logical methodology and tools for specifying and verifying the complex systems. Adoption of formal methods does not give us a guarantee of a priori correctness, but they do increase the comprehensive understanding of a given system by disclosing inconsistencies, ambiguities, and incompleteness that might be undetected in other methods. The reason for using formal methods is to prevent all possible design and developer errors in the required analysis, and to develop the same system as the initially designed and analyzed system. As autonomous driving systems also need to operate in complex and unstructured environments, formal methods can be used to describe the system’s requirements and validate the system.

Efforts to apply formal methods to traffic accidents and driving have been ongoing. One research applied formal methods to a vehicle control system [18]. In addition, some research took advantage of a formal verification method for platooning autonomous vehicles [19]. However, these efforts tried to apply formal methods only to specific applications, and it was not enough to use for the description of comprehensive driving situations.

We propose a method to describe ’driving’ more clearly using the specifications of the formal method. The term ’driving’ is defined by using the extended Backus–Naur Form (E-BNF) method, which is actively used to describe context-free grammar in the field of computer science. Instead of formal verification, we adopt a metamorphic testing method to find out the problems and errors of the autonomous driving system in advance.

### 1.3. Metamorphic Testing

It is very important to verify the correctness of software in software development. In software testing, an oracle test is a procedure where the output is known and testers can decide whether the output of the program under testing is correct or not. If you know the correct answer, you can judge whether there is an error in the program through the oracle test. However, in certain situations, oracles do not exist or they are too difficult to apply. This is known as the oracle problem [20].

There is a program in which the test oracle cannot exist. If an oracle does not exist, no one knows the exact result of the program, and it is very difficult for you to calculate the result. For example, if you have a program that gets the sum of two or three numbers, even if the input value is a little large, you can calculate the result. However, it is difficult for a person to check the sum of numbers with 10 million digits.

As another example, if you have a program that correctly calculates the value of a trigonometric function, sine, to 1000 digits below the decimal point, it is not easy to check the accuracy of the result. In this case too, there is no test oracle to calculate the sine function. Instead of oracles, the human tester becomes an oracle and can manually check the test results. However, manual prediction and verification of the program’s results are usually very poor in efficiency and increase the cost of testing.

A test technique presented to solve these problems is the ’metamorphic testing’ technique. Metamorphic testing uses the mathematical relations that exist within a given problem. The relationship between the inputs and outputs is called the metamorphic relations. Metamorphic relations allow you to create a partial oracle instead of full test oracle. The method is as follows. Using the special metamorphic relationship between the input value and the output value, the input value **I,** which has already been executed, and the observed output value **O** from that is converted to the new input value **I’** and the predictable output value **O’**. Although, it is not a perfect oracle because it cannot tell if the output is correct or not for any input, but it can be a partial oracle because it can tell us if the relation between outputs is kept.

For example, the sum of 10 million numbers must be constant regardless of the order in which they are added. Since the anomaly is swappable, the method for testing the addition program is as follows. First, you assign a serial number to each of the 10 million numbers, and then you enter the serial numbers 1 to 10 million to obtain the sum. Say **I** is the input according to the series of numbers and **O** is the result of input I. Then, re-enter the series of numbers in a random order. Say the random-order input is **I’**, and the result is **O’**. If the program works correctly, **O** and **O’** should be the same. If the values of **O** and **O’** are different, it becomes clear that the program has an error.

### 1.4. Problem Statement

The log is a written chunk of data generated by a certain system in order to find out what has happened in the run-time of the system. Then, by definition, it is a set of post-event data and is fundamentally a posteriori, so that we can analyze it mostly in an inductive manner. However, this often results in mere meaningless clues to what actually happened in a complex system since the analysis on the log confronts the following two aspects.

The first is a hodgepodge state of generated logs. Each log is generated usually serially by a single-tasking system but often in parallel by a multitasking system of various modules. The temporal mixture of logs, however, does not matter even for the latter if its modules work independently. The analyzer can trivially detect each separate set of logs by just tagging them. The problem arises when they are highly interconnected in the perspective of a meaningful event. The temporal mixture makes it hard for the analyzer to discern which log is relevant to each other for a single meaningful event.

This leads to the second aspect that the log is a set of fragmented information. A log is generated by a specific module at a particular time; it is in itself partial and low-level, especially in a complex and dynamic system. Thus, only an experienced engineer who comprehends the entire system could collect and aggregate the data into a meaningful event based on speculation [21]. Some salient scenarios imagined by the experienced can be tested, and they are so-called oracle tests. However, not all can be done in that manner since no one can define all the problematic situations of every condition.

Our goal is to design a logging system with which we can obtain a thorough view of a situation, shedding light on both expected and unexpected occurrences of various events. Our approach is to take advantage of specifications of the formal method to deductively design a logging system specifically for a complex and dynamic system such as autonomous vehicles. In formal verification, however, it is difficult to avoid the pitfalls of the interpolation and oracle test problems mentioned above.

Instead, we adopt metamorphic testing to quantitatively verify the formal specifications by drawing metamorphic relations needed for MT from the formal specifications. Note that our practical goal is to enable the logging system to operate interchangeably on both a real vehicle and its simulator, since the latter can be used to detect unknown errors in hardware or software, or both, prior to testing the former in an actual road environment. The experimental configurations are also set up to check this capability.

Chapter 2 describes our logging architecture in detail based on formal specifications, and Chapter 3 explains our quantifiable verification method based on metamorphic testing. Chapter 4 illustrates the experimental setup and presents the results, and Chapter 5 discusses the overall framework.

## 2. The Logging Architecture

### 2.1. Formal Specifications of the Driving Situation

According to A Framework for Automated Driving System Testable Cases and Scenarios, defined by the NHTSA [22], a driving test is composed of four major behaviors or elements: Tactical Maneuver Behaviors (TMBs), Operational Design Domain (ODD) elements, Object and Event Detection and Response (OEDR) behaviors, and Failure Mode Behaviors. In this case, Failure Mode Behaviors are a special group for autonomous driving. So, it can be excluded from the general driving situation. As a result, general driving can be performed by constituting the above three types: TMB, ODD, and OEDR. In more general terms, this can be interpreted as the maneuver a driver is going to control, the environment in which you drive, and the events that occur while driving. In other words, driving is performed in the environment while performing the maneuver, and it can be seen that the event is encountered in the middle of the related processing. So, you can log data of these components to find out the situation and analyze the problems.

**Figure 2:**
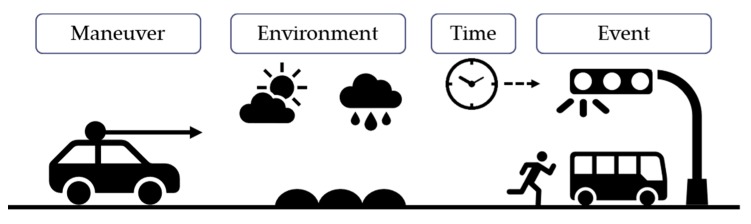
Four components of driving—maneuver, environment, time, and event.

The four components can be classified according to the superset–subset relation based on the formal method. Figure 2 shows four components of driving. This classification is based on the NHTSA classification with some modifications for intuitive usage.

The maneuver mostly follows NHTSA’s classifications for TMB. In the case of maneuvering, it is possible to divide the sections to be traveled while setting the entire travel route—for example, highway drive, low speed shuttle, traffic jam drive, etc. After dividing by sections, it is possible to perform detailed classification according to which driving should be performed in more detail, such as following a car, n-point turns, and low/high speed merge. The driving maneuver eventually appears in the form of a path with speed. In other words, the maneuver to be performed in detail is expressed in the path that the vehicle must follow. So, if we save the path data to a logging system, it may be the same with similar vehicle maneuvers.

In the case of the environment, this includes basic information about the road on which the vehicle must travel and the environmental information such as weather/traffic conditions. Environmental information, for instance, includes the road information, speed limit, and school/construction zone information. This road information can be extracted from a map with location information as data of the Global Positioning System (GPS). It also includes weather and traffic information. These data do not change frequently, and we can record the data manually or use an external image recorder.

Time is the basic component since movement over time is the most elementary data to analyze driving. In addition, changes in the surrounding objects and traffic lights are also recorded based on time.

Events are the most important components for autonomous driving systems. Events can be described by an object and the event that the object generates. So, for the logging system, the objects that can generate the event are listed, and the type of an event generated by the object is described. Events may occur simultaneously, or they may occur in sequence. In order to reproduce an event based on a log, the event object should be recorded. Event objects are classified into six categories, but it can be more detailed or merged depending on the situation. However, together with the event object, the detailed event information generated should be recorded together. Therefore, when storing event information using the logging system, the event object and detailed event information are also recorded.

Applying the formal specification to these driving components can be described as follows. The Extended Backus–Naur form (E-BNF) is used for the formal specification.

<driving> ::= <maneuver> ON <environment> AT <time> [WITH <events>](1)

The meaning of this sentence is that driving is a maneuver in the environment with events that can exist together. The maneuver, environment, time, and events can have the following details. In Table 1, the first stage of each component is presented. The components are refined up to the fourth stage. Details of four-step stages are presented in Appendix A.

Here is an example of the driving situation.

<driving> ::= <maneuver> ON <environment> AT <time> [WITH <events>]            ::= car following ON multilane curves AT 4:13′27″ WITH lead vehicle, stopped(2)

**Figure 3:**
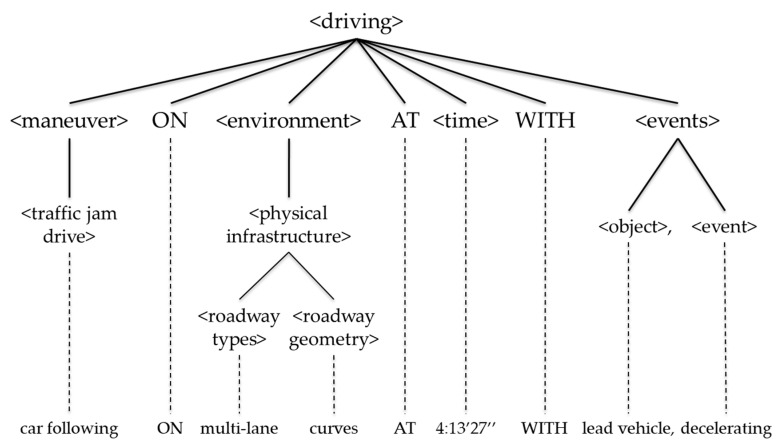
The parsing tree of a driving-state expression.

Such a driving-state expression can be parsed into the following tree as in Figure 3.

By using the formal specification to describe the behavior of ’driving’, it is possible to identify which logging item belongs to which components and, thus, locate an erroneous or error-prone part more clearly.

The autonomous driving system usually consists of perception, planning, and control modules. The maneuver, environment, time, and events are appropriately handled in these three modules to perform autonomous driving. Although they cannot be mapped exclusively, basically, maneuvers will be processed in the planning module, and environment and events will be processed in the perception module. Of course, vehicle control will be done in the control module too. To record how the autonomous driving system responds to environments and events and reacts to maneuvers, it must be able to record detailed information about the perception, planning, and control modules. Therefore, the logging item is based on recording the planning result that determined the maneuver by perception, adding the perception result to the information corresponding to events and the environment.

**Figure 4:**
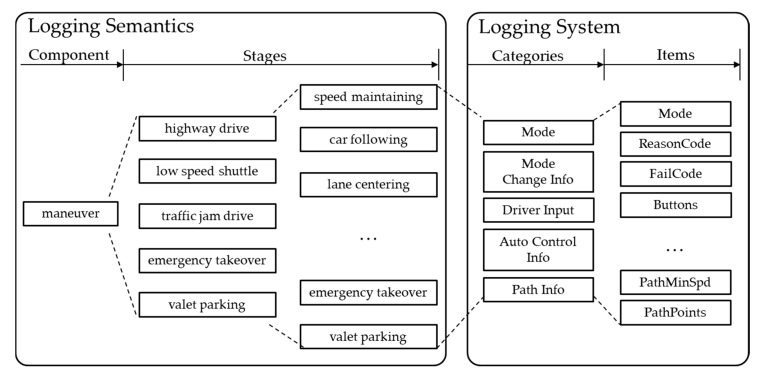
Maneuver component and logging items.

The maneuver determines the movement of the vehicle, that is, the actions of actuators the vehicle should take in a given environment. It selects actions based on the current environment (e.g., highway drive or traffic jam drive), and performs low-level functions such as maintaining speed or following a car. In the autonomous driving system, these functions are performed by low-level actions for routing, path planning, speed profile, and signals. Finally, it results in a steering control value to determine the driving direction of the vehicle, a speed control value to determine the driving speed, and a signal control value to transmit visual information to the nearby vehicles. Therefore, the information of the maneuver can be recorded by recording steer, speed, and signal controls, as depicted in Figure 4.

The logging items for the environment component represents the information about the environment in which the vehicle is driven. This includes information, which needs to be recorded in real time but does not change significantly after being recorded once, such as weather conditions, road geometry, road surface, speed limit, etc. For example, in the case of road geometry, this information indicates which type of road is being driven on, and if a high definition map (HDMap) is used, the road geometry can be inferred from its location. An HDMap is necessary for the self-driving system.

**Figure 5:**
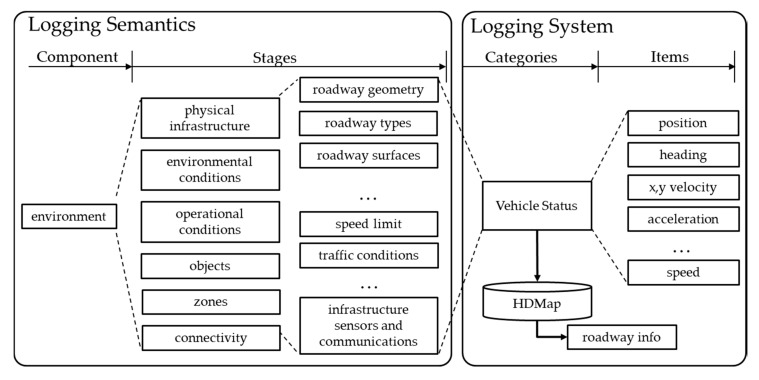
Environment component and logging items.

In addition, for the information not included in the map (e.g., temperature or traffic conditions) but not needed to be recorded quickly, either manual recording or separate image storing can be used for testing instead of automatic recording of the logging system, which records at every moment. Logging items related to the environment is based on position, direction, and matching indices for the map, which can be used to retrieve all data such as road geometry, roadway types, roadway surfaces, and/or other optional information from the map, as depicted in Figure 5.

**Figure 6:**
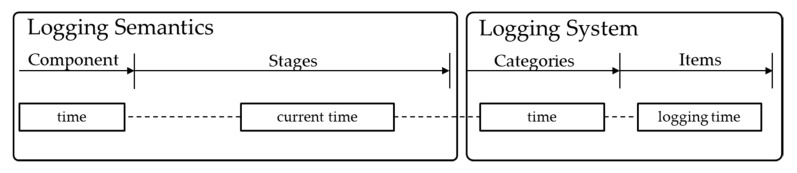
Time components and logging items.

The time component records current time of logging. Time has a simple value, but it is the most important information for detailed logging item analysis. This is because all logging items are recorded with time so that you can start problem analysis based on time. The time is recorded by logging time as shown in Figure 6.

**Figure 7:**
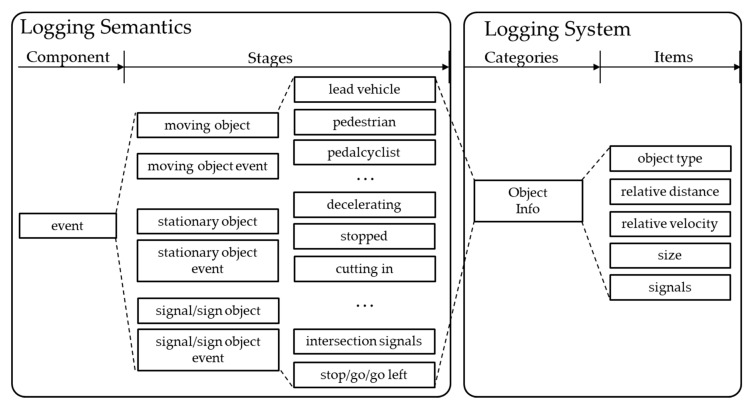
Event components and logging items.

In the case of events during driving, an object that makes the event must be stored because the event occurs according to when the object changes. There are three types of objects: objects with relative distances, fixed objects, and signaling objects that control traffic on the road. Objects of any type can appear at the same time. In addition, it is necessary to record object types and their relative distances. Therefore, the object type, relative distance, relative velocity, and signals are recorded together, as depicted in Figure 7.

**Figure 8:**
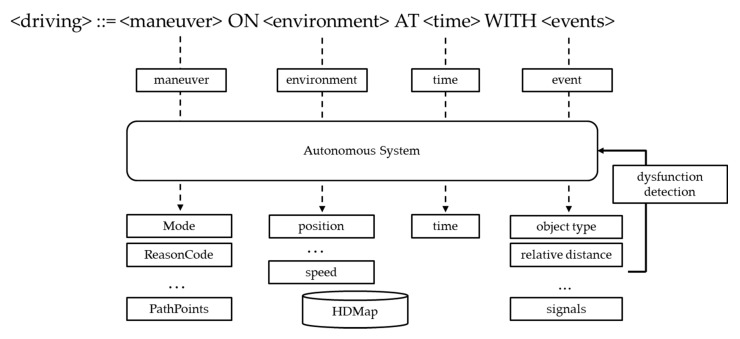
Summary of driving data logging items.

Put together, the components for driving and the subordinate logging items for efficient recording can be summarized as in Figure 8. Driving is the act of performing a maneuver in the environment, at a time, and when events can occur. Thus, the key elements of driving such as the maneuver, environment, time, and events must be recorded. Based on the information, malfunction or dysfunction or latent errors can be detected via logical and mathematical analyses described later.

### 2.2. The Logging Data Structure

Based on formal specifications for driving, the logging system records four components: maneuver, environment, time, and event. Each component has multistep stages. Note that the actual logging data are items, as in Table 1, which are aggregated into categories. These categories are mapped to stages of components from formal specifications described above. By separating formal specifications and logging units, various stages of each driving component can have independent and recurring logging data such as acceleration, target speed, etc.

Maneuver Components
  - Mode: This category includes the current driving mode of the autonomous vehicle, the reason code for entering the current driving mode, and the reason code for generating an approximate error when a system error occurs. The first thing to analyze from the driving information is to check the current driving mode and the reason for the mode change.
  - Mode Change Info: This is for the change in autonomous driving mode. It is important to record driving mode changes because an autonomous vehicle changes its movement according to the driving mode. The previous mode right before the current mode is also recorded. If there were many mode changes in autonomous driving, knowing which mode the vehicle is in before the current stage greatly helps in data analysis.
  - Driver Input: This category stores handling, accelerator, brake information, gear information, steering torque, and so on. With this information, an inspector can see what the driver control was at the time of logging.
  - Control Info: In this category, vehicle control information is stored. Target steering, target speed, and target acceleration are stored. Target acceleration/brake pedal position also can be stored depending on the vehicle speed control type. Turn-signal control information (i.e., left/right turn signal) is also stored. Based on this information, the intention of the autonomous system can be identified.
  - Path Info: Path info stores the driving path generated by the path planning module. The autonomous system drives the vehicle based on this path.
Environment Component
  - Vehicle Status: In this category, the running status of the vehicle is recorded. The current position and heading of the vehicle are recorded by default. The velocity and acceleration along the vertical and horizontal axes of the vehicle are also recorded together. Target speed and current speed during autonomous driving are stored too.
Time Component
  - Time: This category represents the time of logging. The logging time interval can be adjusted as needed. We performed logging every 10 or 20 ms to minimize the loss of very short-term driving information. In future work, this time category could have siblings of more than one child item to represent the temporal context of other components.
Event Component
  - Object Info: This category stores the relationship with objects in the vehicle’s path. This can be used to determine how the vehicle responded to a frontal obstacle.

The configuration of data for a logging item may vary case by case, but it must include the basic components of driving defined by the formal specification. This makes it easier to identify problems with the autonomous driving system. Details of logging items stored for autonomous driving are shown in Table 2.

## 3. Quantifiable Verifications

### 3.1. Metamorphic Relations

In the previous chapter, we used the formal specification to define the behavior and components of driving. Logging items were selected to record key information about the component. The basic data needed to analyze a problem with an autonomous system are recorded in the logging system by definition. However, verification of formal specifications often fails due to the interpolation problem in the practical implementation. Thus, our approach is to convert the logical relations for formal verification to mathematical relations (i.e., metamorphic relations) that can be compared and examined in a quantitative manner.

Metamorphic relations represent consistency of change between input and output values of a certain function. For example, the larger the steering angle is, the greater the change in the heading direction is. This kind of consistency is called an MR. In addition, each log item has aspects such as input and/or output values of a specific behavior of the system. For instance, ’Heading’ can be viewed as an output against ’TargetSteer’ as an input, which is, in turn, an output against ’PathPoints’ as an input. Thus, there are multiple MRs between combinations of each log item.

The important point is its verification method. It is not on the exact output value, like oracles to drive at or decelerate to a certain speed, but on the consistent relationship of output values generated with consistently transformed input values. This often needs hundreds of, even thousands of, experiments to check violations in the consistency. It means that a consistent logical relationship defined in our formal specification in E-BNF can be converted to a quantifiable relationship that is able to be examined without specific oracle values.

Accidents in intelligent dynamic machines with various sensors, like autonomous vehicles, could arise from complex chain reactions between sensors and AI software, even to absent-minded pedestrian behaviors, not to mention trivial device or program errors since many modules are involved in each other. It means they are more vulnerable to the interpolation or oracle test problems. Thus, this quantifiable verification over the problems could substitute formal verification.

### 3.2. Analysis Methods

There are two kinds of analyses based on the proposed logging architecture. One is to analyze predefined test cases from prioritized MRs a bit similar to oracle tests. The other is to explore already examined test cases again from the perspective of different MRs. For this, the logging system pushes all the items into the log database, and then they can be retrieved as different categories for another stage. This is the ultimate goal of our system; however, it needs the former as a prerequisite. Thus, the current analysis method is only for predefined MRs.

As described in Chapter 4 with examples, the analysis method first defines a specific MR. This MR would typically provide one or more relations for which arithmetic comparisons can be done. In particular, the operands of a comparison are each set of results for the same experiment with slightly transformed input values.

In order to analyze a system using a predefined MR, first you need to have an environment where you can run a large number of experiments. This is not necessarily a large system, but rather an environment that can provide various inputs and receive the results. When MR is used to find problems in the test system, it is not usually possible to find the problem with one or two experiments; hundreds or thousands of experiment times are needed by changing the input data and environment.

If such a large number of experiments is physically impossible, the simulation environment is constructed and the experiment is performed. For example, in order to test autonomous vehicles, there are ways in which many vehicles are mounted and checked in various environments after installing the system, but there are problems of cost and time. Therefore, only a part of the experiment using real cars is carried out, and many tests are actively used for simulation.

After establishing the experimental environment, we define the components necessary for the experiment using the formal specifications defined in Chapter 2. Defining components using formal specifications has the advantage of defining only essential elements. Next, we determine how to change MR-related inputs in the experimental environment, and then we conduct a large number of experiments to confirm whether MRs are violated. For metamorphic testing, formally defining MRs has been tried in various ways [23].

For instance, the first stage of the Maneuver component, ’Traffic jam drive’, could be selected due to its frequency in urban areas, then it can be decomposed into the second-stage maneuvers of ’car following’, ’maintain speed’, ’follow driving law’, ’obstacle avoidance’, and so on. Those maneuvers need obstacles for the Event component. Target MRs can be defined using the specific maneuvering functions and corresponding obstacles (e.g., lanes, traffic signal, other vehicles) as transformable input values. The MRs should be defined to have arithmetically comparable relationships rather than having the exact expected result values. It is more preferable to have relationships not between results but between sets of results. Then, the established MRs as above can be tested in a quantitative manner, overcoming the interpolation problem and oracle-test problem. This refinement process can be described as below:

(step1) maneuver<driving>::= <maneuver> 
::= <traffic jam drive> ::= “car following” 
(step 2) environment::= “car following” ON <environment> ::= “car following” ON <physical infrastructure> ::= “car following” ON <roadway geometry><roadway types>::= “car following” ON “straightaways”, “urban”
(step3) time::= “car following” ON “straightaways”, “urban” AT “time”
(step4) events::= “car following” ON “straightaways”, “urban” AT “time” WITH <events>::= “car following” ON “straightaways”, “urban” AT “time” WITH <object>,<object event>::= “car following” ON “straightaways”, “urban” AT “time” WITH “lead vehicle”, “stopped”

There are several ways to confirm MR violation. For example, if the results of each experiment can be compared individually, it can be confirmed immediately if the MR is violated at least once in the experiments. In this case, a detailed analysis can be performed immediately. On the other hand, sometimes, confirmation of MR violation is necessary by the average value. In that case, MR violation can be admitted only after the target number of experiments is completed.

Thus, all we have to do is to conduct hundreds or more than thousands of repetitive experiments to generate log items. After all the experiments, we keep tallies of them as operands of the comparison. If the result of the comparison does not fit the predefined one, then the test would reveal latent error of an autonomous driving system.

## 4. Experimental Results

### 4.1. System Configurations

**Figure 9:**
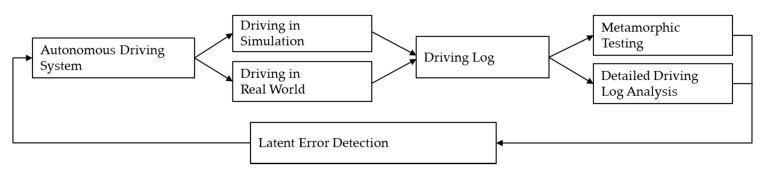
Test sequence of an autonomous driving system.

To show the usability of driving data, we constructed two experimental environments. One is a driving test using a simulation, and the other is a driving log analysis technique based on an actual vehicle. Both of these test environments used an autonomous driving system that we implemented. The test sequence is shown in Figure 9.

**Figure 10:**
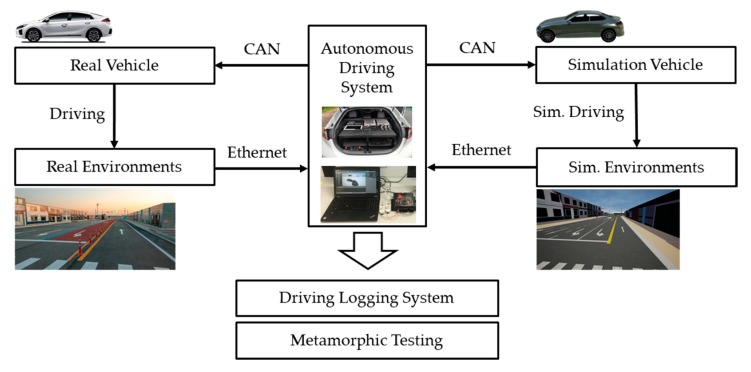
Experiment setting—real vs. simulation.

The experiment environment is shown in Figure 10. On the left is a real car with an autonomous drive system on a test track. On the right is a system configured to test the same autonomous driving system on the simulator. In the simulation test, vehicle, driving environment, and recognition system were implemented in the simulator, and the planning and control systems used the same system mounted on the actual test vehicle. The simulator was based on the CARLA (Car Learning to Act) simulator, which was developed for the purpose of autonomous driving research [24]. We implemented ethernet and controller area network (CAN) connection modules to communicate with our autonomous driving system in the CARLA simulator. In addition to providing the interface, the system maintained the same data format so that the system installed in the vehicle could be directly used in the simulator.

By constructing these two environments, it is advantageous to use a simulator for testing various situations, for repeated testing, and for testing using a real vehicle to check the actual driving responsiveness. In addition, when the simulator is used alone, the result may be different from the response of the actual vehicle. However, by constructing such a real vehicle simulation testing environment, the difference between the simulator and the actual vehicle can be checked, and the gap can be reduced. In this study, the tests and the methods performed for each of these two environments will be described.

**Figure 11:**
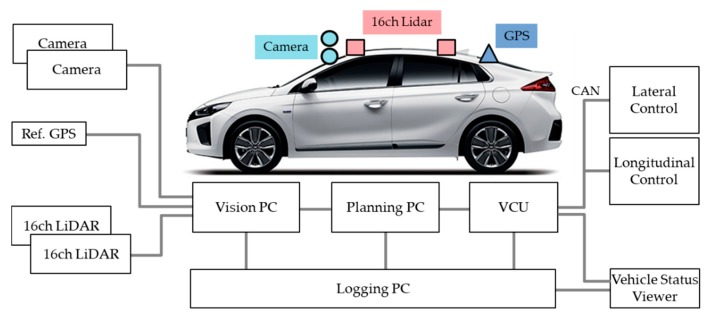
Vehicle hardware configuration.

A test vehicle for autonomous driving is shown in Figure 11. The vehicle was a small sedan, fully electric vehicle. Two cameras and two 16ch LiDAR sensors were used as vehicle sensors. It was also equipped with a high-performance GPS for obtaining reference information. To control the vehicle, control commands were issued via a Controller Area Network (CAN).

The autonomous vehicle system that we implemented used three PCs and one embedded board. The perception module working on Vision PC recognized the vehicle’s current position and surrounding obstacles. Vision PC received sensor data by linking to GPS, cameras, LiDAR, etc., and it calculated the current position and direction of the vehicle using the received sensor data. It also detected obstacles with 360 degree coverage. It estimated the location, direction, and speed of current obstacles. The planning module was run on Planning PC. It calculated the destination of the autonomous vehicle and a global route that must be traversed to reach its destination. In addition, a local path to be followed in detail to travel the global route was calculated and generated in real time. The VCU running on the embedded board received the local path from the Planning PC and generated the actual control command to perform the control. The control commands generated by the VCU were target steering angle and target acceleration. In addition, the VCU managed the current status of the vehicle, terminated autonomous running when there was a problem, manually operated the vehicle in a system failure mode as necessary, and automatically stopped the vehicle. The Logging PC recorded the logging data. The logging data included the items we described in Chapter 2. Logging data files used the ASCII format for easy data reading and understanding.

**Figure 12:**
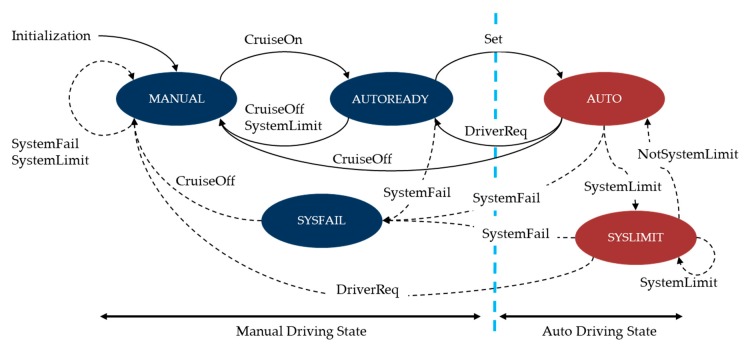
Driving states, modes, and transition diagram.

An autonomous vehicle has five modes internally in two driving states. The two driving states are manual driving state and auto driving state. Manual driving state is a mode in which the driver operates the steering wheel, brake, and acceleration directly. In auto driving state, an autonomous driving system operates the vehicle by itself. There are five modes in these two states: MANUAL, AUTOREADY, and SYSFAIL modes are in the manual driving state, and AUTO and SYSLIMIT modes exist in the auto driving state. The transition between each mode occurs according to the driver’s action and the conditions of the autonomous vehicle. The transition between each mode is shown in Figure 12.

### 4.2. Metamorphic Test Examples

In this section, we show examples of MT for the autonomous driving system and logging data used at that time. First, we identify the MRs. Next, we show testing input data and resulted output data for testing. Finally, we show the MT results.

#### 4.2.1. MT_1_: Stop Regardless of the Obstacle Order

When driving on the road, obstacles may exist in front of the vehicle. At this time, the vehicle shall be capable of stopping based on the nearest obstacle, even if several obstacles exist. For example, when there are several obstacles, the vehicle must be able to stop based on the nearest obstacle to avoid collisions. In other words, even if there are obstacles in any order, the autonomous driving system should react based on the closest obstacle. This property is described in MR_1_.

- MR_1_: if D = {*x* | *x* is a set of an obstacle’s relative distance}, then the ego-vehicle must stop at min(D).

When there is a set of obstacles on the driving path, the ego-vehicle should always stop based on obstacles with the least relative distance regardless of the order in which the obstacle is inputted.

In order to do the metamorphic test based on MR_1_, the input D_o_ was set as follows: some obstacle vehicles were stopped at 10 m intervals on the driving path straight along the vertical axis. The ego-vehicle autonomously drives the path in the positive direction of the vertical axis. In this situation, the ego-vehicle should stop at min(D_o_) regardless of the order or number of obstacles. Then, it satisfies MR_1_.

- Input D_o_: D_o_ = {the vertical axis position of obstacles}
- Expected output of D_o_: ego-vehicle should stop at min(D_o_)

Various test input sets were specified based on MR_1_ by mixing the order of elements, adding a certain level of noise, or adding elements. If the test system satisfies MR_1_, the result should show that the ego-vehicle always stops at min(D*_i_*_={1,2,3}_) regardless of test inputs. Test inputs are shown in Table 3.

**Figure 13:**
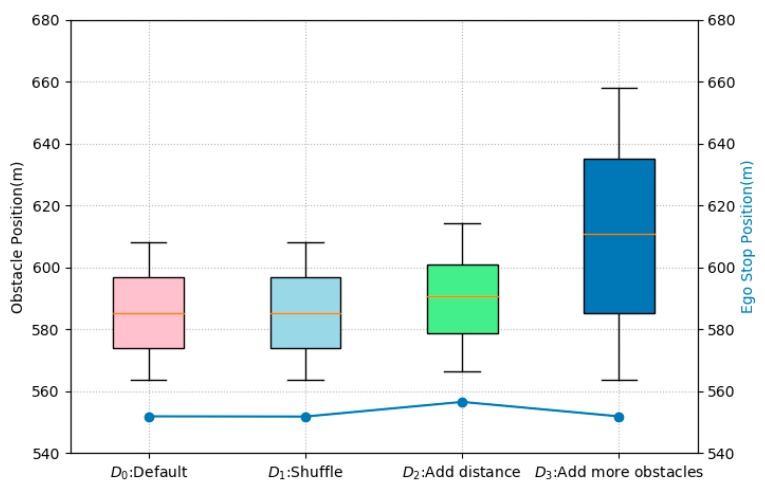
Ego-vehicle stop with obstacle position.

Experiments were conducted using a simulator. Five vehicles were placed at intervals of 10 m between *y*-coordinates, 565 and 610, as initial obstacles. This initial situation was set to Input set D_o_. For metamorphic testing, input set D_1_ changed the order of vehicles based on input set D_o_, Input set D_2_ to which a random value between 2 and 7 was added, and Input set D_3_ to which 10 vehicles were added. Repeated experiments were performed for each input, and a total of 1800 or more experiments were performed. Figure 13 shows the results of the experiment.

In Figure 13, the horizontal axis represents the input set. Input sets D_1_, D_2_, and D_3_ changed based on the basic input set D_o_ and MR_1_. The left vertical axis and boxplot show the distribution of obstacle vehicles. Input sets D_o_ and D_1_ have the same variance because they are different only in ordering. In D_2_, a random value between 2 and 7 was added to the distance value. Both the minimum and maximum values were larger. In D_3_, the addition of 10 vehicles resulted in an increased variance. The right vertical axis and the line show the ego-vehicle stopping position. The same change as the change of the minimum value regardless of the variance of obstacles is shown. This can be interpreted as satisfying MR_1_.

#### 4.2.2. MT_2_: Stop Before the Stop Line

The autonomous driving system used in experiments was programmed to stop just before the stop line when the traffic light was red. The operation order of this function is as follows.

1. Perception: traffic light recognition (red) and ego-vehicle localization;
2. Planning: calculation of the remaining distance to the stop line based on map data;
3. Control: calculation of the target speed based on remaining distance and braking control.

An important factor for the vehicle to stop before the stop line is its current speed and distance to the stop line. If we simplify the problem by making the vehicle speed constant, the only factor would become the distance to the stop line. Basically, when the vehicle speed is constant and there is a red light on a traffic signal, the farther the vehicle is from the stop line, the more likely the vehicle will stop before the stop line. The following is the description about this relation as MR_2_.

- MR_2_: if Input set D_o_ and D_1_ exist, then |R_o_| <= |R_1_| holds.
- If Input set D_o_ = {*x*, *y* | *x* is ego-vehicle speed, *y* is a distance to the stop line} is given, the system returns Output set R_o_ = {*x*, *y* | *x* is the ego-vehicle speed and must be zero, *y* is a distance to the stop line and must be positive}.
- If Input set D_1_ = {*x*, *y*+*d* | *x* is the ego-vehicle speed, *y* is a distance to the stop line, *d* is a positive value} is given, the system returns Output set R_1_ = {*x*, *y* | *x* is the ego-vehicle speed and must be zero, *y* is a distance to the stop line and must be positive}.
- |R_o_| and |R_1_| mean the number of elements in R_o_ and R_1_, respectively.

First, reference data, Input set D_0_, was generated. Then, a test input set D_1_ satisfying the property of MR_2_ was generated. The test input set was generated by adding an additional distance *d* to the distance of *y* in Input set D_o_. According to MR_2_, even if the distance *y* is changed to *y* + *d* for the same speed, *x*, the number of stops before the stop line R_1_ must be the same as or larger than R_o_. So, |R_o_|≤|R_1_| means the systems is correct. On the contrary, |R_o_| > |R_1_| means that there is an error in the system. Test inputs are shown in Table 4.

**Figure 14:**
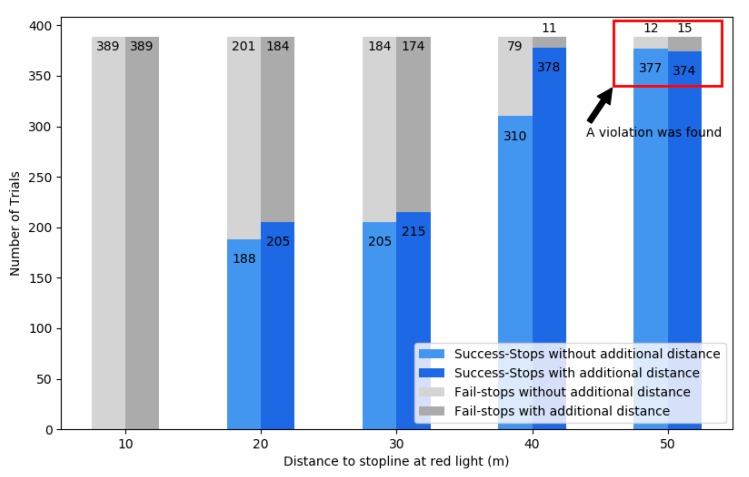
Stop behind the stop line in the red light test.

For testing the reference input, the vehicle was driven at a constant speed (25 km/h or 35 km/h), and the traffic light turned red when the distance from the vehicle to the stop line was within a preset distance (50, 40, 30, 20, and 10 m). Based on this input, the additional distance, *d*, was selected with a random, uniform distribution. Test results are shown in Figure 14.

The horizontal axis is the distance between the vehicle and the stop line when the red signal is turned on, and the vertical axis is the number of attempts. The light-gray bar is the number of failures to stop with the reference input, the dark-gray bar is the number of failures to stop given with the additional distance, the light-blue bar is the number of successful stops with the reference input, and the dark-blue bar is the number of successes stops when the additional distance is given. The experiment was carried out by changing the distance to the stop line from 50 to 10 m in 10 m units. Each set of experiments for a specific distance to the stop line was performed about 380 times, and the total number of experiments was about 3820.

According to MR_2_, the dark-blue bar must be greater than or equal to the light-blue bar, that is, |R_o_| ≤ |R_1_|. Four sets of experiments showed this relationship. However, an exception was found at 50 m: the dark-blue bar was smaller than the light-blue bar, which means a violation of MR_2_. By this verification, we found a latent error that is seldom disclosed.

To identify the cause of the error, we carried out a process in the following order: (1) identify the experiment that had the problem, (2) check the detail log using the logging data defined above, (3) in the detailed log, confirm which part of the system had the problem, and (4) precise analysis is performed about the corresponding part.

In search of the cause, we confirmed that the vehicle failed to stop in the experiment with the additional distance. Then, we checked the detailed logs for the experiment, and the log showed no problem with the distance between the vehicle speed and the stop line. However, the target acceleration output was $0.1\;m/s^{2}$, not $0.0\;m/s^{2}$, and it was confirmed that the vehicle continued to move forward and did not stop at the stop line due to the nonzero target acceleration value. Based on this, it was found that there had been an error in the target acceleration calculation module.

Due to cost and time problems, it is difficult for developers to conduct thousands of interactive tests. This case, in particular, was pretty hard to reveal in usual repetitive experiments since the target speed calculation module worked well without any problems in other tests, and the 50 m test also showed problems very occasionally. The MT detected this scarce phenomenon by performing experiments of a reasonable repetition size (under 400 times), which violated a specified MR based on our logging architecture.

#### 4.2.3. MT_3_: Avoiding Obstacles with a Minimum Margin

While driving, it is common to see vehicles on shoulders or on the side of the road. In some situations, it may be necessary to avoid these obstacles even if the vehicle partially overlaps the next lane. There are many things to consider when autonomous vehicles are driving in such an environment.

Basically, avoidance is done by having a sufficient distance from obstacle vehicles, but the more distance you have, the more you move to the next lane, which increases the risk of accidents. Therefore, it is necessary to decide whether to avoid the obstacle considering the location of the vehicles, and in avoidance, the vehicle bypasses obstacles with a minimum extra distance. In other words, when deciding whether to avoid the location of an obstacle, vehicles should be considered, which shows that there is a relation between an avoidance maneuver and obstacles. The simplest relationship between the two factors is that the larger the avoidance space is, the greater the possibility of performing the avoidance gets. In other words, if the area of the ego-lane occupied by the obstacle vehicle is smaller, the possibility of avoidance is increased. This aspect of the avoidance maneuver can be defined as an MR as below.

- MR_3_: if Input set D_o_ and D_1_ exist, then |R_o_| <= |R_1_| holds.
- If Input set D_o_ = {**p, q** | **p** is ego-vehicle position, **q** is an obstacle position} is given, the system returns Output set R_o_ = {**p** | **p** is the ego vehicle position and the distance between **p** and the final position in path must be less than or equal to the preset Euclidean distance *e* (e.g., *e* = 1.0 m)}.
- If Input set D_1_ = {**p**, **q**+**d** | **p** is the ego-vehicle position, **q** is an obstacle position, **d** is a positive random value and greater than |**d_o_**|} is given, the system returns Output set R_1_ = {**p**| **p** is the ego-vehicle position and the distance between **p** and the final position in path must be less than or equal to the preset Euclidean distance *e* (e.g., *e* = 1.0 m)}.
- |R_o_| and |R_1_| mean the number of elements in R_o_ and R_1_, respectively.
- Note that **p**, **q**, and **d** are coordinates in two-dimensional space and written in bold.

**Figure 15:**
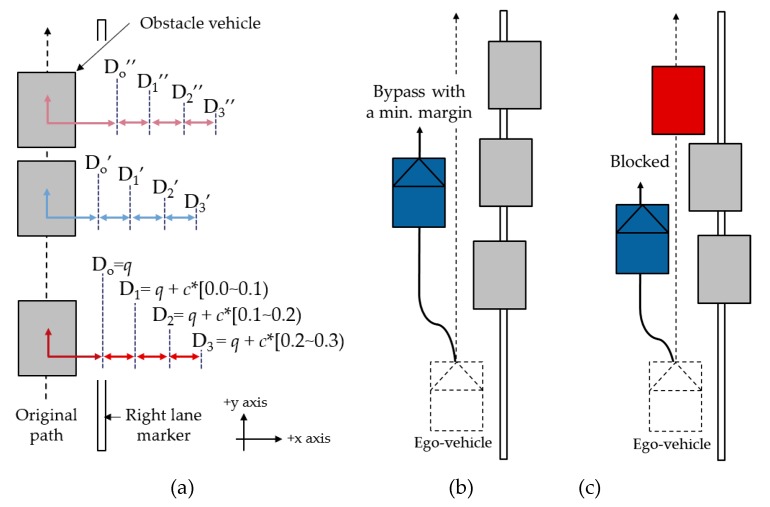
(**a**) Avoidance test setup and two scenarios of the test: (**b**) Bypass with a min. margin vs. (**c**) Blocked.

First, reference data, Original input set D_0_, was generated. Then, test input sets D_1_, D_2_, and D_3_ satisfying the property of MR_3_ were generated sequentially by adding a positive random value. If the test system satisfies MR_3_, the result should show that the number of trials of the ego-vehicle arriving at the final point in Input set D_0_ must be equal to or less than the number of trials with D*_i_*_={1,2,3}_ regardless of any positive random values **d**. Test setup and two scenarios of the test are depicted in Figure 15.

Test inputs are shown in Table 5. The test input set was generated by adding an additional positive random value **d** to **q**, the position of obstacles, based on the input set D_o_. According to MR_3_, even if the distance **q** is changed to **q** + **d** for the positive value, the number of stops at the final point on path R_1_ must be the same as or larger than R_o_. So, |R_o_|≤|R_1_| means the systems is correct. On the contrary, |R_o_| > |R_1_| means that there is an error in the system.

For testing with the reference input, three obstacle vehicles were used. These three obstacle vehicles were generated on the original path with variance **q** and moved by **d_i={1,2,3}_**. The original path was straight and followed the center of the lane. The lane had lane markers on both sides. A test had one result out of two scenarios: either ’Bypass with a minimal margin’ or ’Blocked’. If all obstacles are generated with sufficient margins, the ego-vehicle bypasses the obstacles. On the other hand, if one of the obstacles does not have enough distance from the path, the vehicle will be blocked. Test setup and two scenarios of the test are shown in Figure 15.

**Figure 16:**
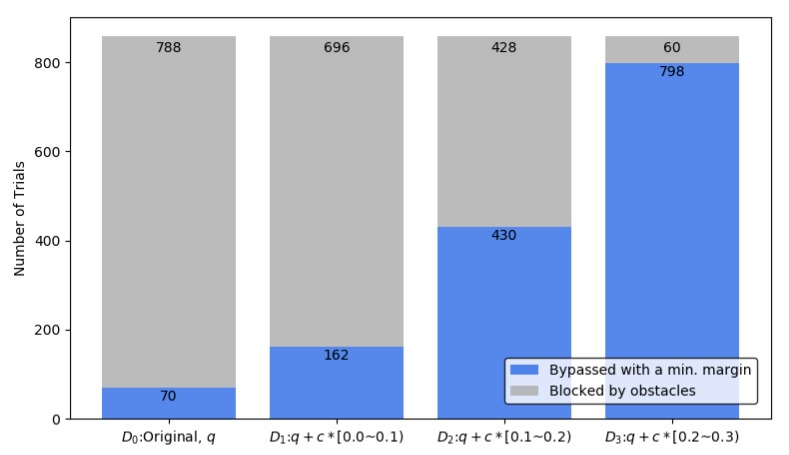
Avoiding obstacles test results.

Based on this input, the additional distance D*_i_*_={1,2,3}_ was selected with a random, uniform distribution. Test results are shown in Figure 16. The maximum value of D*_i_*_={o,1,2}_ was less than the minimum value of D*_j_*_={1,2,3}_ in order to satisfy MR_3_. For each test input set, over 850 tests were conducted and over 3400 tests in total were done. If the system tested in this MT satisfies MR_3_, R_3_ must have the biggest number of trial results of bypassing than any of R_o_, R_1_, and R_2_.

The horizontal axis is the test cases, D*_i_*_={o,1,2,3}_, and the vertical axis is the number of trials. The light-blue bar and the light-gray bar represent the numbers of ’bypassing obstacles’ and ’blocked by obstacles’ results, respectively. The sum of the numbers of light-blue bars and light-gray bars is the same as the total number of trials. The experiment was carried out by selecting a random distance from the original path. Each test case did not overlap each other to be suitable for MR_3_.

According to MR_3_, the light-blue bar of D*_i_*_={o,1,2}_ must be less than or equal to the light-blue bar of D*_j_*_={1,2,3}_, that is, |R_o_| ≤ |R_1_|. The result of this MT shows the relation. So, the test system satisfies MR_3_.

### 4.3. Logging Data Analysis

**Figure 17:**
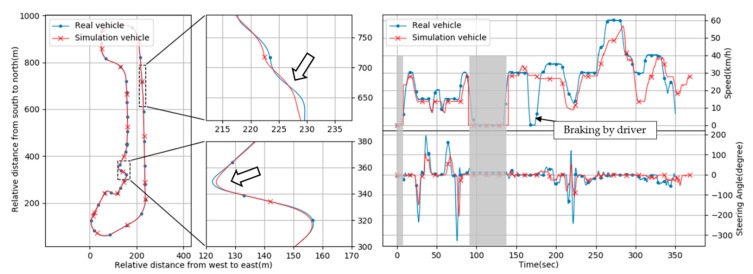
Logging data from real vs. simulation vehicle driving: (**left**) relative driving path, (**right**) speed and steering angle info.

Log data are obtained from test driving of the vehicle based on the logging system described in Chapter 2. Figure 17 illustrates an example of log data. The graphs in Figure 17a show a round driving path; the entire path (left), a lane-change section (top right), and a curve-turn section (bottom right). The horizontal axis is the relative distance from west to east, and the vertical axis is the relative distance from south to north in meters. The trajectories of the real and simulation vehicles are similar in general (left) but slightly different in parts (right). Although it is ideal that the simulation vehicle produced the exact same result as the real vehicle did, it is impossible in practice due to various factors we cannot control, including environmental parameters and insufficient simulation models of all the phenomena including engine dynamics, tire traction, etc. Thus, our practical goal is to make them as similar as possible to the extent that the algorithmic and/or procedural consistency of vehicle behaviors can be compared.

The graphs in Figure 17b show the graphs of the vehicle speed (top) and steering angle (bottom). The upper vertical axis is the ego-vehicle speed (km/h) and the lower vertical axis is the steering angle (degree). The horizontal axis of both graphs is time (s). Note that the dummy data (zero value) was inserted in the simulation vehicle data for plotting in accordance with real vehicle data (the intervals are grayed out in blocks). That area was ignored in the actual comparison. Of course, the real vehicle data were not modified at all. Those graphs show the differences of the real and simulation vehicles more clearly. In the interval from 20 to 80 s, the simulation vehicle generated similar results compared to the real vehicle’s. However, the simulation vehicle produced different results compared to the real vehicle’s from 250 to 320 s. A big difference occurred around 170 s, which was caused by unanticipated braking of the test driver in the real vehicle.

Although the simulation vehicle used in this study did not produce the same results as the real vehicle’s, it generated consistent patterns that were similar, which can be used to detect unknown errors existing in the real vehicle.

**Figure 18:**
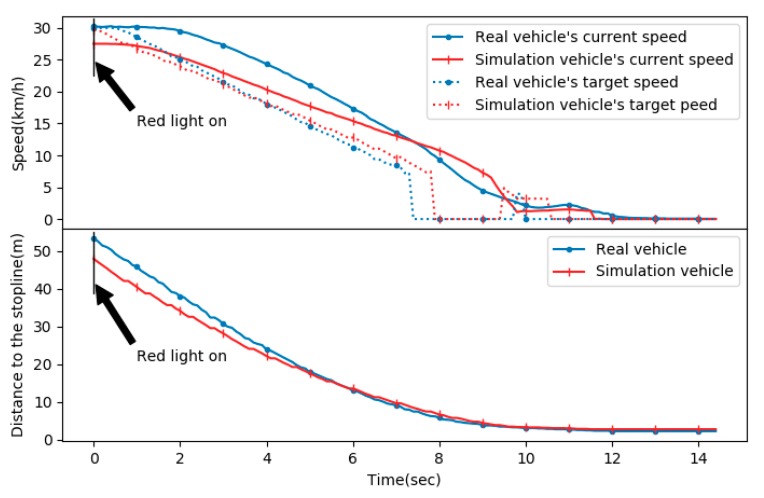
Real vs. simulation: vehicle current and target speeds (**upper**), the distance to the stop line (**lower**).

In order to compare the log data of the simulation and the real vehicle’s driving in more detail, a vehicle-stopping experiment was performed with respect to the traffic light. Figure 18 shows the comparison between the real vehicle’s logging data with the simulation vehicle’s logging data in a red light situation. There are two graphs sharing the horizontal axis representing time (s). The vertical axis of the upper graph is the speed (km/h), and the other one of the lower graph is the remaining distance (m) to the stop line.

In the upper graph, the solid blue line is the real vehicle’s current speed, and the solid red line is the simulation vehicle’s current speed. The dashed blue line is the real vehicle’s target speed, and the dashed red line is the simulation vehicle’s target speed. In the lower graph, the distance to the stop line, the solid blue line is the remaining distance to the stop line of the real vehicle, and the red solid line is the remaining distance to the stop line of the simulation vehicle.

In the two graphs, both the real vehicle and the simulation vehicle showed almost the same results. Especially, in the speed graph, both vehicles’ target speeds were set to 0 km/h at about 8 s, and then the target speed increased to about 5 km/h at around 10 s. Although the detailed values were slightly different, similar control patterns appeared when the real vehicle and the simulation were controlled by the test system. As a result, it can be seen that the vehicle test through the simulation showed a pattern similar to the real vehicle test, which means that the test of the autonomous vehicle control system through the simulation is meaningful.

## 5. Conclusions

In this work, we designed a log analysis framework to be useful in testing an autonomous driving system. Complex interactions of well-designed/tested units and algorithms could produce minor errors, dysfunction, and even severe malfunction. Fortunately, there is a source of clues to the cause of problems in a practically working machine, which is, of course, its log data generated by each module on the fly. In the autonomous vehicle industry, many log recording systems such as the EDR have already been adopted (even mandatorily in some regions). However, the heap of log data per se does not tell us what will happen before visible malfunction or accidents.

Therefore, we proposed a logging architecture based on formal specifications, providing a priori relationships between modules and/or data and a log analysis method based on quantifiable verification of a posteriori relationships, which overcomes the interpolation and oracle test problems. Using this framework, testers can define unanticipated test cases, results of which can be checked via tangible log data from a working system by a number of automatable repetitive tests.

For formal specification, we used E-BNF to describe the logical relationships for an autonomous driving system and NHTSA’s framework as a standard model for the relationships. Then, we adopted MT for quantifiable verification of the specified relationships, which were used as the source of MRs. To examine the effectiveness of our approach, we implemented the logging architecture in both real and simulation vehicles and defined three test cases based on three MRs, respectively, which included both longitudinal and lateral behaviors of the vehicle.

Thousands of similar experiments with slight differences have shown us that our autonomous vehicle had a latent error that could cause accidents on a given day. This did not frustrate us, but it gave us confidence that an autonomous vehicle with our logging architecture could be examined on a concrete basis of systematic logs and the corresponding analysis method.

The experiments of three MRs and MTs, however, are not sufficient to demonstrate practical effectiveness of our framework. In particular, despite our guideline to procedurally draw MRs and corresponding MTs from formal specifications expressed via E-BNF, it is still cumbersome and needs some expertise. This should be alleviated by a semi-automatable method.

Note that the current grammar in E-BNF lacks confidence levels of sensor values necessary to cope with not only binary (positive/negative) decision points but also uncertainty for perception and planning systems. Due to the logical and deterministic nature of formal languages like E-BNF, our formal specification does not embrace fuzzy or stochastic situations. Thus, a probabilistic or stochastic model such as the Markov chain would be introduced to our deterministic model.

Although we did not pursue value-by-value similarities between real and simulation vehicle behaviors, the discrepancy between them should be bounded by some given extent so that the simulation result can be a reasonable substitute for the actual driving test. For this, the bounding method could formally be defined. We will be working on this in the near future.

We can extend our relationship model by considering the standardized safety test catalogues, such as the New Car Assessment Program (NCAP) established by various governments, especially for fatal situations like crashes. We expect it will increase the relevance of our framework to the industry.

## Figures and Tables

**Table 1:** The first stage of driving components.

<driving>	::=	<maneuver> ON <environment> AT <time> [WITH <events>]		
<maneuver>			::=	<highway drive>
				| <low speed shuttle>
				| <traffic jam drive>
				| <emergency takeover>
				| <valet parking>
<environment>			::=	<physical infrastructure>
				[, <environmental conditions>]
				[, <operational constrains>]
				[, <objects>]
				[, <zones>]
				[, <connectivity>]
<time>			::=	“time”
<events>			::=	<object>, <object event>
				{<object>, <object event>}

**Table 2:** Details of logging items for an autonomous driving system.

Component	Category	Item	Description
Maneuver	Mode	Mode	System’s current working mode
		ReasonCode	Reasons for entering the current mode
		FailCode	Reasons for system failure
	Mode Change Info	Buttons	Buttons pressed by the driver
		StateIn	Previous system mode
	Driver Input	APS	Accelerator pedal position value
		BPS	Brake pedal position value
		SAS	Steering angle sensor value
		GearPos	Transmission gear position (P, R, N, D)
		OBD2Spd	OBD2-based vehicle speed
		PushBrake	Push brake? On/Off
		SteeringTorque	Steering torque value
	Auto Control Info	TargetSteer	Target steering command
		TargetSpeed	Target speed command
		TargetAccel	Target acceleration command
		Target Signal	Left/Right turn signal command
	Path Info	PathLen	Path length
		PathMinSpd	Minimum speed in path
		PathPoints	Start, mid, last points in path
Environment	Vehicle Status	Longitude	GPS longitude
		Latitude	GPS latitude
		Heading	GPS heading
		LongVel	Longitude velocity
		LatiVel	Latitude velocity
		LongAccel	Longitude acceleration
		LatiAccel	Latitude acceleration
		WheelSpd	Vehicle rear wheel average speed
		VelCurr	Current speed of the vehicle
Time	Time	Logging Time	Current logging time
Event	Object Info	ObjectType	Object type
		Drel	Relative distance to object
		Vrel	Relative velocity to object
		Size	Object size
		Signals	Traffic light signals

**Table 3:** Test inputs for stopping regardless of obstacle order.

Inputs	Description	Expected Result
D_o_	Original input set	Stop before min(D_o_)
D_1_	Shuffle the elements in D_o_	Stop before min(D_1_)
D_2_	Add random noise (2 m < *n* < 7 m) to the elements in D_o_	Stop before min(D_2_)
D_3_	Add more elements in D_o_	Stop before min(D_3_)

**Table 4:** Test inputs for stopping at the stop line.

**Inputs**	**Description**	**Expected Result**
D_o_	Original input set	|R_o_|
D_1_	New input set D_1_ = {*x*, *y*+*d* | *x*, *y* in D_o_, *d* is positive value}	|R_1_| (Satisfying |R_o_| ≤ |R_1_|)

**Table 5:** Test inputs for the avoidance test.

Inputs	Description	Expected Result
D_o_	Original input set D_o_ = {**p***,* **q** | **p** is ego-vehicle position, **q** is obstacle position, **q** = *c* * [0.0~0.4) }	|R_o_|
D_1_	Input set D_1_ = {**p**, **q**+**d_1_** | **p**, **q** in D_o_, **d_1_** is positive random value, **d_1_** = *c* * [0.0~0.1) }	|R_o_| <= |R_1_|
D_2_	Input set D_2_ = {**p**, **q**+**d_2_** | **p**, **q** in D_o_, **d_2_** is positive random value, **d_2_** = *c* * [0.1~0.2) }	|R_o_| <= |R_1_| <= |R_2_|
D_3_	Input set D_3_ = {**p**, **q**+**d_3_** | **p**, **q** in D_o_, **d_3_** is positive random value, **d_3_** = *c* * [0.2~0.3) }	|R_o_| <= |R_1_|<= |R_2_|<= |R_3_|

*c is the preset constant (e.g., road width, vehicle width).

## Appendix A

In this appendix, the detailed components of driving defined in this study are shown. Driving, defined in this study, consisted of four elements: maneuver, environment, time, and events. Maneuver used with the binary operator ON represents tasks performed during operation, and the environment after operator ON describes the environment in which the task is performed. Events used with the Unary operator WITH represent events that occurred during driving. This detailed component is summarized based on the NHTSA framework.

**Table A1:** The driving components.

<**driving**>	::=	<maneuver> ON <environment> AT <time> [WITH <events>]

**Table A2:** The 1st and 2nd stages of <maneuver>.

<**maneuver**>	::=	<highway drive>
		| <low speed shuttle>
		| <traffic jam drive>
		| <emergency takeover>
		| <valet parking>
<highway drive>	::=	“maintain speed”
		| “car following”
		| “lane centering”
		| “lane switching/overtaking”
		| “obstacle avoidance”
		| “follow driving law”
		| “low/high speed merge”
<low speed shuttle>	::=	“maintain speed”
		| “car following”
		| “lane centering”
		| “lane switching/overtaking”
		| “obstacle avoidance”
		| “follow driving law”
		| “stop at destination”
		| “n-point turn”
		| “route planning”
		| “parking”
<traffic jam drive>	::=	“maintain speed”
		| “car following”
		| “lane centering”
		| “lane switching/overtaking”
		| “obstacle avoidance”
		| “follow driving law”
		| “low speed merge”
<emergency takeover>	::=	“takeover request”
		“takeover response”
<valet parking>	::=	“maintain speed”
		| “car following”
		| “obstacle avoidance”
		| “follow driving law”
		| “parking”

**Table A3:** The 1st and 2nd stages of <environment>.

<**environment**>	::=	<physical infrastructure>
		[, <environmental conditions>]
		[, <operational constrains>]
		[, <objects> ]
		[, <zones> ]
		[, <connectivity> ]
<physical infrastructure>	::=	<roadway geometry>
		[, <roadway types>]
		[, <roadway surfaces>]
		[, <roadway edges>]
<environmental conditions>	::=	<weather>
		, <weather-induced roadway conditions>
		, <particulate matter>
		, <illumination>
<operational constrains>	::=	<speed limit>
		, <traffic conditions>
<objects>	::=	<signage>
		, <roadway users>
		, <non-roadway user obstacles/objects>
<zones>	::=	“geo-fencing”
		| “traffic management zones”
		| “school zone”
		| “construction zones”
		| “regions/states”
		| “interference zones”
<connectivity>	::=	“V2V”
		| “traffic density information”
		| “remote fleet management system”
		| “infrastructure sensors and communications”

**Table A4:** The 3rd stage of <environment><physical infrastructure>.

<**physical infrastructure**>	::=	<roadway geometry>
		[, <roadway types>]
		[, <roadway surfaces>]
		[, <roadway edges>]
<roadway geometry>	::=	“straightaways”
		| “curves”
		| “hills”
		| “lateral crests”
		| “corners”
		| “negative obstacles”
		| “lane width”
<roadway types>	::=	“divided highway”
		| “undivided highway”
		| “urban”
		| “rural”
		| “parking”
		| “multi-lane”
		| “single-lane”
		| “4-way/2-way stop”
		| “merge lanes”
<roadway surfaces>	::=	“asphalt”
		| “concrete”
		| “mixed”
		| “brick”
		| “dirt”
<roadway edges>	::=	“line markers”
		| “temporary line markers”
		| “shoulder(paved or gravel)”
		| “shoulder(grass)”
		| “concrete barriers”
		| “curb”
		| “cones”

**Table A5:** The 3rd stage of <environment><environmental conditions>.

<**environmental conditions**>	::=	<weather>
		, <weather-induced roadway conditions>
		, <particulate matter>
		, <illumination>
<weather>	::=	“temperature", ( "snow" | "wind" | <rain> )
<weather-induced roadway conditions>	::=	“standing water”
		| “flooded roadways”
		| “icy roads”
		| “snow on road”
<particulate matter>	::=	“fog”
		| “smoke”
		| “smog”
		| “dust/dirt”
		| “mud”
<illumination>	::=	“day”
		| “dawn”
		| “dusk”
		| “night”
		| “street lights”
		| “headlights”
		| “oncoming vehicle lights”

**Table A6:** The 3rd stage of <environment><operational constrains>.

<**operational constrains**>	::=	<speed limit>
		, <traffic conditions>
<speed limit>	::=	“minimum speed limit”
		, "maximum speed limit”
<traffic conditions>	::=	“minimal traffic”
		| “normal traffic”
		| “bumper-to-bumper/rush-hour traffic”
		| “altered(accident, construction, …)”

**Table A7:** The 3rd stage of <environment><objects>.

<**objects**>	::=	<signage>
		, <roadway users>
		, <non-roadway user obstacles/objects>
<signage>	::=	<signs>
		| <traffic signals>
		| “crosswalk”
		| “railroad crossing”
		| “stopped bus”
		| “construction signage”
		| “hand signals”
<roadway users>	::=	<vehicle types>
		, "stopped vehicle”
		, "moving vehicles”
		| “pedestrians”
		| “cyclists”
<non-roadway user obstacles /objects>	::=	<animals>
		| “shopping carts”
		| <debris>
		| “construction equipment”
		| “pedestrians"
		| “cyclist”

**Table A8:** The 4th stage of <environment><objects><signage>.

<signs>	::=	“stop”
		| “yield”
		| “pedestrian”
<traffic signals>	::=	“flashing”
		| “school zone”
		| “fire department zone”
		| “etc”

**Table A9:** The 4th stage of <environment><objects><roadway users>.

<vehicle types>	::=	“cars”
		| “light trucks”
		| “large trucks”
		| “motorcycles”

**Table A10:** The 4th stage of <environment><objects>< non-roadway user obstacles/objects>.

<**animals**>	::=	“dogs”
		| “cats”
		| “deer”
		| “etc”
<debris>	::=	“pieces of tire”
		| “trash”
		| “ladders”
		| “etc”

**Table A11:** The 1st stage of <time>.

<**time**>	::=	“time”

**Table A12:** The 1st and 2nd stages of <events>.

<**events**>	::=	(<object>, <object event>)
		{, (<object>, <object event>) }
<object>	::=	“lead vehicle”
		| “side/back vehicle”
		| “pedestrian”
		| “pedalcyclist”
		| “animal/debris/dynamic object”
		| “signals/signs”
<object event>	::=	“decelerating”
		| “stopped”
		| “accelerating”
		| “turning”
		| “cutting out”
		| “parking”
